# Retraction: Genome-wide association mapping for high temperature tolerance in wheat through 90k SNP array using physiological and yield traits

**DOI:** 10.1371/journal.pone.0286776

**Published:** 2023-06-14

**Authors:** The PLOS ONE Editors

The *PLOS ONE* Editors retract this article [1] because it was identified as one of a series of submissions for which we have concerns about authorship, competing interests, and peer review. We regret that the issues were not addressed prior to the article’s publication.

MARR did not agree with the retraction. HGMDA, MN, YZ, AU, AS, and AQ either did not respond directly or could not be reached.
